# The changing epidemiology of human type 2 diabetes–associated atherosclerosis: Pathophysiological mechanisms and emerging treatment possibilities

**DOI:** 10.1111/joim.70118

**Published:** 2026-06-12

**Authors:** Dania Al‐Sharify, Jiangming Sun, Andreas Edsfeldt

**Affiliations:** ^1^ Department of Clinical Sciences Malmö Lund University Malmö Sweden; ^2^ Department of Cardiology University Hospital of Skåne Lund/Malmö Sweden; ^3^ Wallenberg Centre for Molecular Medicine Lund University Lund Sweden

**Keywords:** atherosclerosis, cardiovascular complications, genetics, imaging, therapies, type 2 diabetes

## Abstract

Type 2 diabetes (T2D) is a major global health concern strongly associated with atherosclerosis and subsequent macrovascular complications. These complications are the leading cause of death among T2D patients. Despite a decline in cardiovascular events over the last decade, individuals with T2D still have an approximately doubled risk compared to those without diabetes. This shows an urgent need for therapies targeting biological processes specific to T2D‐associated atherosclerosis. Nevertheless, more research is needed to identify exactly which processes can be targeted therapeutically. Current therapies either target lipid metabolism or inflammation, two processes commonly considered important in T2D‐associated atherosclerosis. However, more recent human plaque tissue studies show no differences in plaque levels of lipids nor inflammatory markers, possibly reflecting improved clinical treatment strategies. Other distinct differences in plaque tissue composition in T2D have been put forward, including thin fibrous caps and large necrotic cores. Moreover, T2D may influence several biological processes affecting both plaque formation and progression (such as oxidative stress and efferocytosis). These mechanisms could potentially also be targeted to prevent atherosclerotic cardiovascular complications. This review focuses on the shifting epidemiology of T2D‐associated cardiovascular complications, as well as biological changes in T2D plaques, and how these changes can guide future clinical approaches to further reduce atherosclerotic complications.

## Graphical abstract

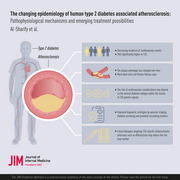

## Introduction

Type 2 diabetes (T2D) is a major global health burden affecting over 500 million people. Furthermore, its prevalence is on a continuous rise, projected to reach more than 1 billion by the year 2050 [1]. T2D is linked to a range of complications affecting various organs, with vascular complications (micro‐ and macrovascular) being the most severe. Cardiovascular disease (CVD)—including coronary artery disease (CAD), ischemic stroke, and peripheral artery disease (PAD)—is a major cause of morbidity and mortality among individuals with T2D. As management of cardiovascular risk factors has improved, the incidence of cardiovascular complications has declined. However, it remains significantly higher in individuals with T2D, indicating that the diabetes‐specific mechanisms underlying cardiovascular complications are not targeted efficiently using current therapy. In this review, we aim to provide insight into the changing epidemiology of T2D‐associated CVD, the biological changes characterizing T2D‐associated atherosclerosis, and how these changes can be identified using imaging techniques and/or targeted therapeutically.

## Cardiovascular complications now and then

### T2D‐associated cardiovascular complications

**Fig. 1:**
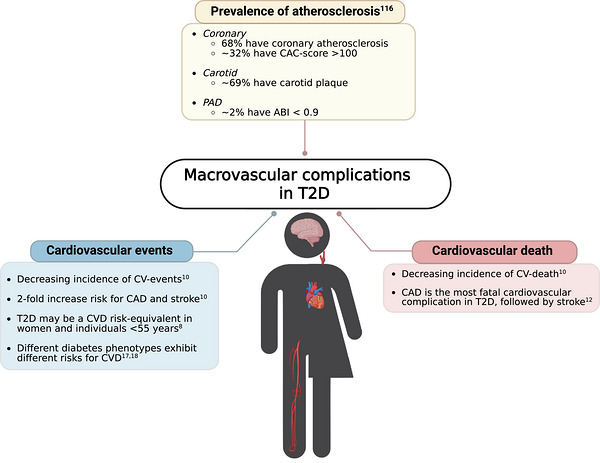
Type 2 diabetes and the risk of macrovascular complications. An illustration summarizing the latest understanding of the epidemiology of diabetes‐associated cardiovascular disease. The yellow box includes information about the prevalence of atherosclerosis in different vascular beds in asymptomatic subjects with known diabetes. The blue box includes information about the risk of cardiovascular events in subjects with type 2 diabetes compared to subjects without diabetes, whereas the red box refers to cardiovascular death. ABI, ankle brachial index; CAC, coronary artery calcification; CAD, coronary artery disease; CV, cardiovascular; CVD, cardiovascular disease; PAD, peripheral artery disease; T2D, type 2 diabetes. Created in BioRender. Al‐Sharify, D. (2026) https://BioRender.com/unb52tg.

T2D has been proposed to be a very strong risk factor for atherosclerosis‐associated CVD, including PAD, CAD, and carotid atherosclerosis (Figure 1) [2]. Having T2D was previously considered to be an equivalent risk factor as having suffered from a myocardial infarction [3, 4]. Yet, a meta‐analysis published in 2009, including 45,000 individuals followed for up to 25 years, showed that the risk of CAD (both fatal and non‐fatal) was approximately 43% lower in individuals with diabetes with no previous myocardial infarction compared to individuals without diabetes who previously suffered from a myocardial infarction [5]. Another meta‐analysis demonstrated that the increased risk of CAD and ischemic stroke due to diabetes was greater among women and those under the age of 60 [6]. The duration of diabetes was also of importance; the risk of ischemic stroke increased by 3% for each diabetes‐year, and the risk tripled when the duration reached ≥10 years [7]. These results were further corroborated by Zhao et al. [8], reporting that diabetes diagnosis may be a CVD risk equivalent in certain populations—such as in women and individuals younger than 55 years.

**Fig. 2:**
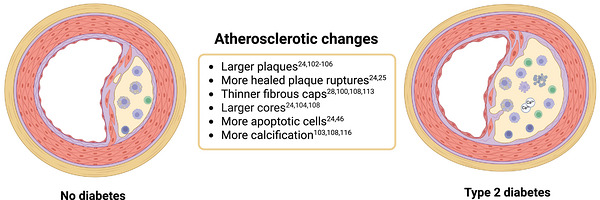
Morphological characteristics of type 2 diabetes–associated atherosclerosis. An illustration of the differences in plaque morphological features between subjects with and without type 2 diabetes. Plaques from patients with type 2 diabetes have been described to have thinner fibrous caps, larger cores, more dead cells, and more calcification. Plaques from individuals with type 2 diabetes are generally also larger and more frequently show signs of plaque ruptures. Created in BioRender. Al‐Sharify, D. (2026) https://BioRender.com/unb52tg.

It is also well recognized that T2D is a significant risk factor for cardiovascular death. Individuals with T2D have at least two‐fold higher risk of dying from cardiovascular complications, compared to individuals without diabetes [2, 6, 9, 10, 11]. Similarly, a systematic literature review, including four million individuals with T2D, identified CAD as the most fatal cardiovascular complication in T2D, followed by stroke [12]. This highlights not only the frequency but also the severity of CVD complications in T2D.

### Decreasing incidence of T2D‐associated CVD

With advances in the management of T2D, both cardiovascular event rates and cardiovascular mortality rates have declined. In Sweden, between 1998 and 2014, the rate of diabetes‐associated cardiovascular death decreased by 46% and hospitalization for CVD dropped by 44% [10]. This likely reflects our clinically improved and intensified medical treatment strategies to target common risk factors, such as hypertension, dyslipidemia, and glycemic control. In support of the importance of targeting cardiovascular risk factors, a study involving 1.5 million individuals demonstrated that individuals >65 years of age with diabetes and no major cardiovascular risk factor (hypertension, smoking, high hemoglobin A1c [HbA1c], albuminuria, or hypercholesterolemia) did not have a higher risk of stroke events compared to individuals without diabetes. However, individuals <55 years of age with all risk factors present had a six to eight‐fold increased risk for cardiovascular events compared to matched controls without diabetes [13]. This suggests that common cardiovascular risk factors act synergistically to increase the risk of cardiovascular complications in diabetes.

The observed trends in T2D‐associated CVD over time may also be influenced by improved diabetes screening strategies, considering the prevalence of undiagnosed diabetes or prediabetes among individuals who have suffered from a myocardial infarction [14]. An early identification of diabetes and prediabetes may be of particular importance considering the high risk of suffering from a recurrent event also among individuals with prediabetes [15].

## Phenotypic, genetic, and epigenetic heterogeneity in T2D‐associated CVD

In recent years, it has been shown that adult‐onset diabetes could be classified into different subtypes based on clinical parameters. Interestingly, it has also been suggested that the risk to suffer from cardiovascular complications may be greater in some of these clinical subtypes. In 2018, Ahlqvist et al. [16] described five distinct subtypes of adult‐onset diabetes (severe autoimmune diabetes, severe insulin‐deficient diabetes [SIDD], severe insulin‐resistance diabetes [SIRD], mild obesity–related diabetes [MOD], and mild age–related diabetes [MARD]) based on age at diabetes diagnosis, body mass index (BMI), HbA1c, HOMA2‐B, HOMA2‐IR, and GADA. Even though no strong associations between the five clinical phenotypes and cardiovascular complications were observed in that study, a recent prospective study including >19,000 individuals with diabetes showed that the SIRD phenotype was most strongly associated with myocardial infarction [17]. Another study by Xue et al. [18], using two independent cohorts, described five diabetes subtypes based on four clinical parameters: HbA1c, age at diabetes onset, BMI, and estimated glomerular filtration rate (eGFR). By following >30,000 individuals with T2D over a median time of 11.7 years, they showed that the clinical subtype with renal dysfunction carried the highest risk of vascular events, followed by a cluster with poor glycemic control and a cluster with severe obesity.

In addition, genetic and epigenetic heterogeneity in T2D has also been shown to contribute to CVD risk. Using genome‐wide association studies (GWAS) from over 2.5 million individuals, T2D risk loci were grouped into eight clusters with distinct profiles of cardiometabolic trait associations. Of these, an obesity‐related cluster of T2D signals was more strongly associated with risk of developing CAD and PAD, whereas a beta‐cell dysfunction cluster—characterized by a positive association with proinsulin—demonstrated a protective effect against CAD and ischemic stroke [19]. Furthermore, using blood‐based genome‐wide DNA methylation analysis, Schrader et al. [20] highlighted epigenetic heterogeneity across clinically defined diabetes subtypes in relation to CVD risk and showed that high SIRD‐methylation risk score (MRS) and MARD‐MRS were associated with an increased risk of future macrovascular complications, whereas SIDD‐MRS and MOD‐MRS were associated with a reduced risk. Taken together, these studies indicate that specific genetic and clinical phenotypes of T2D may need to be considered when predicting CVD risk.

## Atherosclerosis

Atherosclerosis is a chronic inflammatory disease characterized by the formation of atherosclerotic plaques in large and medium‐sized arteries. Atherosclerosis is initiated by subendothelial retention and modification (i.e., oxidation) of low‐density lipoproteins (LDL). The oxidized LDL (oxLDL) induces an inflammatory response, leading to an influx of inflammatory cells (in particular, monocytes and T‐cells) into the plaque [21]. The monocytes will differentiate into macrophages, engulf oxLDL, and become lipid‐loaded foam cells. These foam cells will eventually undergo apoptosis, leading to the formation of a necrotic core. As a counterbalancing factor to the inflammatory process, vascular smooth muscle cells (VSMCs) will produce extracellular matrix (ECM) proteins (especially collagens), which will form a fibrous cap on top of the plaque and make the plaque less prone to rupture [21, 22]. Atherosclerosis is a common condition. According to a Swedish study including >25,000 individuals without known CAD (50–64 years of age), approximately 42% of all individuals had atherosclerosis in the coronary arteries [23]. However, most of these atherosclerotic plaques will remain asymptomatic, whereas some become obstructive or cause acute ischemic events due to plaque rupture or erosion.

### T2D‐associated atherosclerosis

Morphological studies performed on coronary plaques support that individuals with T2D have a greater plaque burden and more frequent plaque ruptures compared to individuals without diabetes [24, 25]. The exact mechanisms underlying plaque ruptures remain largely unknown, but both imaging and histological plaque tissue studies have shown that the phenotype of the plaque seems to be of great importance [26, 27]. Interestingly, a histological ratio—reflecting the balance between vulnerable components (neutral lipids, macrophages, and intra‐plaque hemorrhage) and stabilizing plaque components (collagen and VSMCs)—has been shown to be a strong independent predictor of future cardiovascular events, in particular among individuals with T2D [27, 28].

**Fig. 3:**
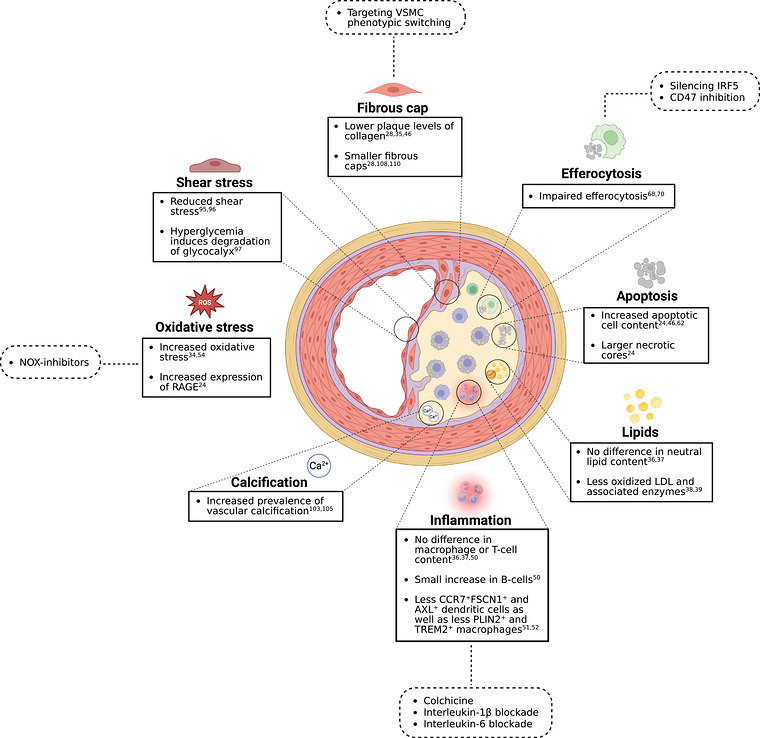
Type 2 diabetes and atherogenesis. Summary of biological pathways/processes that may influence atherosclerotic plaque formation and progression in T2D. The dashed‐lined boxes include therapeutical targets. CD47, cell of differentiation 47; IRF5, interferon regulatory factor 5; NOX, NADPH oxidase; RAGE, receptor for advanced glycation end product; VSMC, vascular smooth muscle cell. Created in BioRender. Al‐Sharify, D. (2026) https://BioRender.com/unb52tg.

Several potentially important biological processes underlying aggravated atherosclerosis in T2D have been put forward based on murine and in vitro studies [29], but here we focus on biological changes observed in human T2D‐associated atherosclerosis (summarized in Figs. 2 and 3).

### Accumulation and modification of lipids

A crucial first step in plaque formation is the retention of circulating lipoproteins, which bind to proteoglycans in the intimal part of the arterial wall [30]. In T2D, the circulating lipoprotein profile is commonly characterized by high levels of triglycerides and low levels of high‐density lipoproteins [31]. Moreover, diabetes is associated with increased concentrations of modified LDL, such as small dense LDL and glycated LDL. Modified LDL particles are atherogenic and more susceptible to oxidation, thereby contributing to increased oxidative stress [32, 33]. Furthermore, insulin resistance is known to increase the circulating levels of free fatty acids (FFAs) due to enhanced lipolysis. These FFAs increase reactive oxygen species (ROS) generation, which, in turn, activates the transcription factor nuclear factor kappa B (NFkB), leading to upregulation of several pro‐inflammatory genes and may contribute to plaque progression [34].

Considering these changes, the accumulation of modified lipids has been suggested to be a key factor contributing to both plaque formation and plaque vulnerability in T2D. Earlier studies have supported this notion, showing a greater plaque area stained positive for oxLDL in plaques from patients with T2D [24, 35]. However, more recent carotid plaque studies from our group and others have not been able to confirm more accumulated lipids in plaques from patients with T2D [36, 37]. Furthermore, our group showed that plaque levels of oxLDL and lipoprotein‐associated phospholipase A2 (an oxLDL‐associated lipase, which hydrolyzes oxidized phospholipids) were significantly lower in plaques from patients with T2D [38, 39]. In line with this, Kathir et al. [40] showed that the lipid oxidation profile was not affected in T2D plaques. These studies indicate that the amount of lipids and modified lipids has been reduced over time, potentially due to intensified lipid‐lowering treatment strategies in T2D.

Interestingly, a more recent study using near‐infrared spectroscopy (NIRS) showed that the pool of accumulated lipids in coronary arteries was not a strong marker of vulnerable plaques in diabetes [41, 42]. Although the latter study did not separate T1D from T2D, these findings suggest that, in addition to lipids, other factors need to be targeted to further reduce the risk of plaque rupture among individuals with T2D who are already receiving statin treatment.

### The local inflammatory process

Both atherosclerosis and T2D are associated with systemic low‐grade inflammation, at least partly mediated by interleukin‐6 (IL‐6) [43]. Therefore, inflammation has commonly been considered a key mechanism for an accelerated CVD in T2D [44, 45]. Earlier histological studies showed a greater inflammatory cell infiltrate, both T‐cells and macrophages, in coronary and carotid plaques from individuals with T2D [24, 35]. In line with this, greater NFkB activity and higher levels of pro‐inflammatory cytokines have been described in plaques from individuals with T2D, indicating not only a greater presence of inflammatory cells but also increased inflammatory activity [35, 46]. Other transcription factors, such as activator protein‐1 and interferon regulatory factor 5 (IRF5), which affect the transcriptional state of monocytes and macrophages, have also been implicated in the increased inflammatory activity previously observed in T2D plaques [47].

However, the association between T2D‐associated inflammation and cardiovascular complications has been questioned in epidemiological studies [48, 49]. Furthermore, human carotid plaque studies from the Athero‐Express (*n* = 1455) and the Carotid Plaque Imaging Project (*n* = 194) biobanks could not show a greater presence of macrophages nor higher levels of chemokines or cytokines in plaques from individuals with T2D [36, 37]. These findings were also supported by a recent single‐cell RNA‐sequencing study comparing the transcriptional state of >69,000 CD45^+^ human plaque cells [50]. In the study by Durán et al., the authors only observed a small increase in B‐cells in plaques from individuals with T2D. Interestingly, though, the authors did observe significantly fewer *CCR7^+^ FSCN1^+^* and *AXL* ^+^ dendritic cells as well as *PLIN2* ^+^ and *TREM2* ^+^ macrophages, which are central in foam cell formation [51, 52]. Even though inflammation is a key factor in plaque formation and vulnerability, these findings indicate that enhanced inflammatory activity is no longer a major biological factor distinguishing T2D atherosclerosis, at least not among individuals with advanced atherosclerotic disease. Moreover, the phenotype of atherosclerotic plaques seems to gradually change over time, also in the general population. According to another histological carotid plaque study from the Athero‐Express, a trend toward a more stable plaque phenotype—with fewer lipids and infiltrating macrophages—was observed over time. The underlying cause of these temporal changes remains unknown but could again potentially be due to an intensified statin treatment [53].

### Oxidative stress

Oxidative stress—defined as an overproduction of the harmful ROS and impairment of the antioxidant mechanisms—has also been suggested to be a key factor in diabetes‐associated atherosclerosis. This redox imbalance induces pro‐inflammatory cytokines and chemokines and promotes atherogenic processes, such as endothelial dysfunction, leukocyte infiltration, and cell apoptosis [54]. An increased oxidative stress in diabetes could be mediated by hyperglycemia, insulin resistance, or dyslipidemia. In a high glucose milieu, the mitochondrial production of the free radical superoxide rises, leading to an inhibition of the key enzyme glyceraldehyde 3‐phosphate dehydrogenase (GAPDH) and the activation of several stress pathways, which leads to the formation of advanced glycation end (AGE) products. When the AGE products bind to their receptor, RAGE, ROS production and epigenetic changes are induced, favoring a pro‐inflammatory outcome [34, 54].

In a human setting, studies, including both coronary and carotid plaques, showed that plaques from patients with T2D had more RAGE expression compared to those from patients without diabetes [24]. Increased plaque levels of RAGE have also been associated with more inflammatory cells and NFkB activity [35]. Moreover, studies using diabetic *ApoE*‐null mice have shown that RAGE is associated with increased plaque formation as well as progression and that inhibiting AGE formation could reduce plaque progression and promote plaque stability [55, 56]. Apart from AGE and RAGE, the ROS‐generative enzyme NADPH oxidase (NOX) has also been suggested to play a potential role in oxidative stress in diabetes‐associated atherosclerosis based on in vitro and in vivo experiments using diabetic ApoE^−/−^ mice [57].

### Apoptosis and core formation

Plaque tissue studies have also demonstrated that T2D plaques have more TUNEL^+^ VSMCs, higher levels of active caspase‐3, and larger necrotic cores compared to plaques from individuals without diabetes [24, 46]. Circulating levels of the apoptosis marker soluble caspase‐3 have also been shown to be higher in individuals with diabetes compared to individuals without diabetes [58]. Furthermore, our group, using the Swedish Malmö Diet and Cancer cohort, found that higher levels of circulating caspase‐3 were associated with increased HbA1c and a greater risk of cardiovascular complications [59]. However, these studies did not distinguish between T1D and T2D, although the majority of cases were T2D.

Accumulation of apoptotic cells is a key feature in both plaque progression and plaque vulnerability [21]. Plaque cell apoptosis is commonly identified at sites of plaque ruptures, and apoptosis has been shown, using ^14^C bomb pulse dating on human carotid plaques, to be associated with faster tissue turnover, indicating a more rapid plaque progression [60, 61]. The underlying cause of the pro‐apoptotic milieu observed in T2D still needs to be explored, but several factors—such as endoplasmic reticulum (ER)‐induced apoptosis of insulin‐resistant macrophages—have been suggested [22]. In support of an important role of ER‐induced apoptosis, Han et al. [62] showed that late‐stage lesions of insulin receptor (Insr) knock‐out mice have a two‐fold increase in apoptotic cell content, potentially due to ER‐induced apoptosis of macrophages, compared to Insr^+/+^ mice.

Besides insulin resistance, hyperglycemia has been found to induce apoptosis of endothelial cell (EC) in vitro [63]. Hyperglycemia is also known to generate AGE products whose accumulation has a detrimental effect on the tissue by inducing apoptosis. Accumulation of AGEs has been observed in macrophages in the interface to the necrotic core of human carotid plaques, co‐localizing with cleaved caspase‐3 [64].

Lastly, T2D‐associated dyslipidemia may also contribute to plaque cell apoptosis, as triglycerides have been reported to induce cell apoptosis [65]. Altogether, several biological processes associated with T2D may contribute to an increased cell apoptosis, which could lead to the accumulation of apoptotic cells and a faster plaque progression.

### Efferocytosis—The removal of apoptotic cells

The necrotic cores (commonly found deep inside the plaque) are dependent on the balance between cell apoptosis and the clearance of apoptotic cells (efferocytosis). A growing body of evidence suggests that the accumulation of apoptotic cells in plaques from patients with T2D could be the result of impaired efferocytosis. Efferocytosis is performed by both professional (macrophages and dendritic cells) and nonprofessional efferocytes (VSMCs and ECs) and is regulated by several signaling pathways, including find‐me, eat‐me, engulfment, and digestion signaling molecules as well as by efferocytosis inhibitors [66]. Inefficient efferocytosis will cause accumulation of apoptotic cells, followed by secondary necrosis, an expanding necrotic core, and thereby plaque progression.

Murine in vitro and in vivo studies have provided evidence for a defective macrophage‐induced efferocytosis caused by features of T2D. Li et al. [67] demonstrated that peritoneal macrophages from obese mice have an increased ratio of saturated:unsaturated fatty acids, causing a defect in the phosphatidylinositol 3‐kinase signaling and, thereby, impaired efferocytosis. Furthermore, using diabetic ApoE^−/−^ mice and cell cultures, Qui et al. [68] demonstrated that hyperglycemia partially reduced apoptotic cell clearance by affecting the efferocytosis‐receptor Mer tyrosine receptor kinase (MerTK) expression on macrophages. When restoring MerTK expression using nanoparticles, the efferocytosis capacity was restored. Another study by Bau et al. [69] showed that high glucose levels caused defective macrophage‐mediated efferocytosis of apoptotic cardiomyocytes, also through an effect on MerTK. Moreover, a recent study by Mao et al. [70]—using peripheral blood monocytes and monocyte‐derived macrophages collected from patients with poorly regulated T2D (defined as HbA1c  ≥ 64 mmol/mol)—showed a decrease in the percentage of monocytes performing efferocytosis in T2D subjects compared to age‐ and sex‐matched controls. They also reported that the number of monocytes did not differ between the populations but that the percentage of classical monocytes (with the greatest efferocytic capacity) was lower in T2D.

Efferocytosis is also dependent on transcription factors regulating the transcriptional state of the cells. One transcription factor that has gained interest in T2D is IRF5, which has been linked to both insulin resistance and impaired efferocytosis [71, 72, 73]. IRF5 promotes macrophage polarization towards a pro‐inflammatory CD11c^+^ transcriptional state with reduced efferocytic capacity. IRF5 has also been associated with hyperglycemia, a vulnerable plaque phenotype, and has recently attracted interest as a potential therapeutic target [73, 74, 75]. However, studies have not found higher protein or mRNA levels of IRF5 in advanced carotid plaques from T2D patients compared to those without diabetes.

Overall, these studies suggest that an impaired efferocytosis capacity may play an important role in T2D atherosclerosis, but further studies are needed to explore if and how T2D affects efferocytosis in human atherosclerosis.

### Fibrous cap formation

Atherosclerotic plaques are separated from the circulating blood by a fibrous cap, mainly consisting of collagen types I and III, which provide structural integrity to the plaque. The thickness of the fibrous cap is an important factor to prevent plaque ruptures, and a thin fibrous cap is considered to reflect a vulnerable, rupture‐prone plaque phenotype [26].

Earlier and more recent histological and biochemical studies have shown that plaques from T2D individuals have lower collagen content and smaller fibrous caps, indicating that the fibrous cap might be of particular importance for atherosclerotic events in T2D [28, 35, 46]. Why plaques from individuals with T2D have smaller fibrous caps remains to be fully understood, but different mechanisms affecting the delicate balance between formation and degradation of the fibrous cap have been suggested.

VSMCs are central in the formation of the fibrous cap. Lineage‐tracing reporter mouse models have indicated that plaque VSMC likely originate from clonal expansion of a smaller number of medial smooth muscle cells, which have migrated into the intima [76, 77]. Human plaque single‐cell RNA‐sequencing studies have shown that these intimal VSMCs are highly plastic and may differentiate into several different phenotypes (i.e., synthetic, macrophage‐like, foam cell‐like, fibroblast‐like, and contractile VMSCs), all with different biological roles [28, 78]. These transcriptional changes are likely induced by factors present in the local plaque microenvironments, including growth factors such as transforming growth factor‐β (TGF‐β).

However, due to transcriptional changes associated with migration of VSMC and the accumulation of CD68^+^ macrophage‐like VSMC in the core regions, the proportion of VSMCs in the atherosclerotic plaques has commonly been underestimated [79, 80]. These transcriptional changes have also been suggested to be more pronounced in diabetes [81].

Most studies comparing carotid plaques from patients with and without T2D have not observed any significant differences in the overall plaque areas stained positive for the traditional contractile VSMC marker, α‐smooth muscle actin [36]. However, when comparing fibrous cap regions specifically, we recently showed that the fibrous caps in plaques from individuals with T2D have less contractile VSMCs [28]. This regional difference could be of great importance as the interplay between contractile (*MYH11* ^high^, *ACTA2* ^high^) and fibroblast‐like (*LUM* ^high^, *COL1A1* ^high^) VSMCs seems to be central in collagen formation. Interestingly, this interplay was halted in human T2D plaques due to a loss of TGF‐β2, leading to impaired collagen production and VSMC differentiation. These findings provide a potential link between impaired fibrous cap formation and the increased risk for plaque ruptures in T2D.

### Degradation of the fibrous cap

Thinner fibrous caps in T2D plaques may also result from increased ECM degradation by proteases such as the matrix metalloproteinases (MMPs). Although several MMPs contribute to fibrous cap degradation, human plaque studies indicate that MMP‐9 is a key protease associated with plaque vulnerability. MMP‐9 has been linked to future events and has also been shown, by our group and others, to be upregulated in the ruptured and unstable plaque regions [82, 83]. Earlier studies also reported higher levels of MMP‐9 and MMP‐2 in plaques from individuals with T2D, along with a greater inflammatory activity [35]. However, more recent plaque studies have not observed higher levels of MMP‐2 or MMP‐9 in T2D [37]. Furthermore, our group recently showed that isolated carotid plaque cells from individuals with T2D had reduced MMP‐2 activity compared to cells isolated from patients without diabetes [28]. This is notable, as MMP‐2 is important for VSMC migration, and reduced MMP‐2 activity may therefore affect VSMC migration and fibrous cap formation. However, future studies are needed to determine the importance of changes in MMP‐induced fibrous cap degradation in T2D.

### Vascular calcification

Vascular calcification is also a common plaque feature, known to be associated with a greater plaque burden as well as with T2D atherosclerosis [84, 85]. Vascular calcification is commonly divided into medial calcification (affecting peripheral arteries) and intimal calcification, which may affect plaque vulnerability depending on location and the calcification pattern [86, 87]. Calcification is considered to be an active biological process dependent on the release of bone‐forming proteins such as osteocalcin and osteopontin as well as osteogenic transformation of VSMC [88, 89]. Even though this VSMC transformation is less well characterized in human plaques, it has been linked to T2D‐associated conditions such as hyperglycemia [90, 91]. Intimal calcification may also be initiated by the release of phospholipid‐rich debris from apoptotic cells in the core regions. The accumulated phospholipid‐rich debris serves to nucleate apatite and could lead to crystal growth [25].

Which specific factors promote vascular calcification in T2D remain unknown but could include cell death, oxidative stress, and hyperglycemia.

### Shear stress

Endothelial shear stress refers to the force exerted by the blood flow on the endothelial layer of the arterial wall. In arterial branches and bifurcations, the shear stress is low or oscillatory, making these sites more susceptible to plaque formation. In contrast, regions with high shear stress are generally protected from atherosclerosis [92]. ECs lining the innermost layer of the arterial wall have mechanosensors located in their apical and basal surfaces as well as at the cell–cell junctions. These sensors react to biomechanical stimuli, such as shear stress, and transduce them into signals that induce transcriptomic and epigenomic changes, thereby modulating various endothelial functions—including EC morphology, inflammation, endothelial‐to‐mesenchymal transition, cell death, permeability, and nitric oxide (NO) production [93, 94]. Diabetes has been shown to be associated with reduced shear stress in the carotid arteries [95, 96]. Moreover, hyperglycemia has been shown to contribute to the degradation of the glycocalyx (a mechanosensor), affecting EC responses to shear stress, resulting in endothelial dysfunction and, consequently, increased permeability and reduced NO production [97]. Moreover, an in vitro study using porcine aortic ECs demonstrated that high glucose levels impair the ECs’ ability to undergo morphological changes in response to laminar flow shear stress, contributing to endothelial dysfunction [98]. In summary, both human and in vitro studies suggest that shear stress alterations may contribute to the aggravated atherosclerotic disease in T2D.

## Imaging evidence for advanced atherosclerosis in T2D

**Fig. 4:**
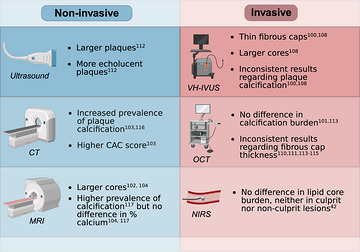
Characterization of type 2 diabetic plaques using invasive and non‐invasive imaging modalities. A summary of imaging studies using the currently available imaging modalities for characterization of type 2 diabetes plaque composition in vivo. CAC, coronary artery calcification; CT, computed tomography; MRI, magnetic resonance imaging; NIRS, near‐infrared spectroscopy; OCT, optical coherence tomography; VH‐IVUS, virtual histology intravascular ultrasound. Created in BioRender. Al‐Sharify, D. (2026) https://BioRender.com/unb52tg.

Advancements in imaging technologies have made it possible to better characterize the morphology of atherosclerotic plaques in vivo, both invasively and noninvasively, using modalities such as ultrasound, intravascular ultrasound (IVUS), optical coherence tomography (OCT), computed tomography (CT), and magnetic resonance imaging (MRI). In the last two decades, an increasing number of imaging studies have been conducted comparing plaque morphology of subjects with and without diabetes, aiming to identify key components next to plaque size, which could be used to identify high‐risk plaques or individuals (summarized in Fig. 4). Unfortunately, a large part of these studies has not separated individuals with T1D and T2D, hence biasing the interpretation of the T2D‐specific patterns. However, imaging studies have repeatedly shown that atherosclerotic plaques in individuals with T2D have a more rapid progression [99, 100, 101] and are larger [24, 102, 103, 104, 105, 106] compared to plaques from individuals without diabetes. Here, we focus on biological differences (core size, fibrous cap, and calcification), which could be identified by current imaging techniques and may also contribute to the increased risk of cardiovascular complications in T2D.

### Core size

Imaging studies comparing plaque core size between subjects with and without T2D generally support the findings from histological studies. In 2010, Esposito et al. [102] used MRI to compare carotid plaque phenotypes between individuals with and without T2D and showed that types IV and V plaques (with lipid or necrotic cores), according to the modified American Heart Association plaque classification [107], were more common among individuals with T2D. Moreover, a more recent MRI study on symptomatic patients from China demonstrated that T2D was associated with a higher prevalence of lipid‐rich necrotic cores in carotid plaques, even after adjusting for conventional clinical risk factors [104]. These findings are also supported by a recent IVUS study, performed on subjects with stable angina pectoris, which showed that the necrotic core volume (both absolute and percentage) was greater in coronary plaques from patients with diabetes [108]. However, studies that have not distinguished between T1D and T2D have shown varying results regarding lipid content and core size [109, 110, 111].

### Fibrous cap

IVUS studies have reported a higher frequency of thin fibrous caps in coronary plaques from individuals with T2D and symptomatic CAD, well in line with the previously mentioned histological studies [28, 100, 108]. Moreover, an ultrasound‐based study conducted in 2007, including 46 subjects with T2D and 51 subjects without diabetes, showed that T2D subjects have more echolucent carotid plaques, indicating less fibrous tissue and potentially more lipids and intraplaque hemorrhage [112]. Even though findings from OCT studies are conflicting [110, 111], a recent large OCT study also observed thinner fibrous caps in T2D plaques, in line with previous IVUS studies [113]. Furthermore, studies analyzing non‐culprit coronary plaques have confirmed that plaques from individuals with diabetes have thinner fibrous caps, particularly in those with poor glycemic control (HbA1c ≥ 64 mmol/mol) [114, 115]. However, these OCT studies did not separate T1D and T2D, which may have affected the results.

### Calcification

Calcification, commonly assessed by CT, is a well‐described marker of atherosclerosis, and studies have provided evidence for an increased prevalence of calcification in T2D coronary plaques [103, 116]. A large population‐based study including 4804 subjects with prediabetes, 2282 with diabetes (diabetes type was not specified), and 27,024 without diabetes, recruited from 6 different Swedish hospitals between 2013 and 2018, showed that diabetes was associated with greater coronary artery calcification [116].

A VH‐IVUS study including 90 patients with stable angina reported a greater dense calcium area in coronary plaques from individuals with T2D [108]. However, in a recent study, yet small in size, no difference in dense calcium area at baseline nor at follow‐up was identified [100]. Results from OCT studies have also been inconsistent. OCT studies including only T2D individuals have not been able to identify a significant difference in calcification burden [101, 113], whereas studies not distinguishing between T1D and T2D have shown a greater calcification in diabetes‐associated plaques [111, 114]. As for carotid plaques, a multicenter MRI study including symptomatic patients (182 with diabetes and 402 with no diabetes) in China found a higher prevalence of calcification in carotid plaques of individuals with diabetes, even after adjusting for clinical risk factors. Yet, when comparing % calcification, no significant difference was observed between the groups [117]. In contrast, another MRI study of carotid plaques conducted in 2017 did not find an association between diabetes and plaque calcification [104].

## Genetics and epigenetics—Is there a link to T2D‐associated CVD?

### Genetic contribution to T2D‐associated CVD

GWAS studies have improved insights into the genetic basis of T2D and its cardiovascular complications, including CAD, ischemic stroke, and PAD [118, 119, 120]. Recent large‐scale GWAS have identified hundreds of associated loci across these conditions (T2D: 611; CAD: 303; ischemic stroke: 45; PAD: 19). Genetic correlation analyses further demonstrated that T2D shared measurable heritability with CAD, ischemic stroke, and PAD, indicating the presence of overlapping biological pathways contributing to both T2D and cardiovascular complications [118, 120, 121]. Genetic susceptibility loci shared between T2D and cardiovascular complications (CAD, ischemic stroke, and PAD) converge on key biological processes underlying metabolic and vascular pathology, including lipid metabolism, inflammation, and ECM remodeling. To identify genetic variants whose effects on vascular outcomes differ between individuals with and without T2D, SNP‐T2D interaction analyses have been conducted across these traits, which identified several diabetes‐dependent risk loci. Among these loci, three (*CDKN2B/CDKN2A*, *SORT1*, and *PDE3A*) showed T2D‐specific associations with CAD, two (*TMEM51* and *TRIQK*) with ischemic stroke, and one (*PTDSS1*) with PAD [121]. In addition, five CAD risk loci (*CDKN2A/2B*, *PHACTR1*, *CELSR2‐PSRC1‐SORT1*, *HNF1A*, and *PCSK9*) and one PAD risk locus (*CCSER1*) have also been reported in T2D [120, 122]. Interaction analyses evaluating whether CAD loci are modified by T2D in the development of subclinical atherosclerosis, including coronary artery calcification (CAC), carotid intima–media thickness (cIMT), and carotid plaque, identified three genes (*ATP1B1*, *ARVCF*, and *LIPG*) associated with CAC and two genes (*ABCG8* and *EIF2B2*) associated with cIMT and carotid plaque, respectively [123]. Together, these GWAS findings may improve risk stratification in T2D and inform targeted interventions to reduce diabetes‐related vascular risk.

### Epigenetic changes linked to CVD in T2D

The field of epigenetics has gained attention in recent years, yet studies about its role in diabetes‐associated atherosclerosis are limited. Epigenetic changes include DNA methylation, non‐coding RNAs, and posttranslational modifications such as histone modifications, altering gene regulation without modification of the DNA sequence [124]. Epigenetic changes are driven by environmental factors such as hyperglycemia, suggesting an important link to diabetes [125]. Hypomethylation of several genes, such as vascular endothelial growth factor B, has been linked to diabetes‐associated CVD [126]. Moreover, in a recently published study, 453 blood‐based DNA methylation sites were shown to be associated with diabetes‐associated macrovascular events. Further, using MRS, including 87 sites, MRS was shown to predict future events in newly diagnosed patients with T2D, and its prediction ability outperformed current cardiovascular risk scores such as SCORE‐Diabetes and Framingham. The study highlighted the potential of epigenetic markers in cardiovascular risk prediction in individuals with T2D [127]. Moreover, a study comparing the genome‐wide profiles of a histone acetylation site on peripheral blood mononuclear cells extracted from humans with advanced atherosclerosis, with or without T2D, did find unique T2D modifications [128]. Interestingly, epigenetic changes have also been hypothesized to explain the glycemic memory phenomenon, also known as “metabolic memory,” which states that hyperglycemia can cause epigenetic changes in the genome that affect gene expression levels even after achieving improved glycemic control. This phenomenon underscores the importance of early and tight glycemic control for prevention of diabetes complications [34].

## Current treatment strategies—What are we targeting

It is evident that intensified medical treatments targeting well‐known cardiovascular risk factors and follow‐up strategies are needed to efficiently reduce the risk of cardiovascular complications among individuals with T2D [13]. However, how we target these risk factors, such as hyperglycemia or circulating lipoproteins, may also be of relevance.

### Glucose‐lowering therapies

Although hyperglycemia has repeatedly been identified as a major risk factor for future cardiovascular events, clinical prospective trials such as the UKPDS, ACCORD, ADVANCE, and VADT, aiming for an intensified glycemic control, did not show a reduction in the frequency of cardiovascular complications [129, 130, 131, 132]. However, extended follow‐up studies from the VADT and the UKPDS later indicated positive effects on cardiovascular events [133, 134], suggesting that improved glycemic control may affect long‐term cardiovascular risk.

Metformin has long been a first‐line treatment in T2D, but its role in preventing cardiovascular complications has been debated. Nevertheless, there are studies indicating that metformin is associated with reduced cardiovascular morbidity and mortality as well as cIMT [135]. Several pathways have been suggested, including reduced NLRP3 inflammasome activation [136]. However, human studies investigating these mechanisms remain limited.

Over the past years, novel glucose‐lowering agents—including glucagon‐like peptide‐1 receptor agonist (GLP‐1RA) and sodium–glucose cotransporter‐2 inhibitor (SGLT2i)—have been introduced to the market, leading to a paradigm shift in the treatment of T2D [137].

Today, there is clinical evidence for GLP‐1RA in reducing atherosclerotic cardiovascular events such as myocardial infarction, stroke, and PAD, but the mechanisms are still under investigation [138, 139, 140]. Preclinical studies using ApoE^−/−^ and LDLr^−/−^ models have presented evidence for GLP‐1RA attenuating plaque progression through inflammatory pathways. Yet, the primary results of a randomized, placebo‐controlled clinical trial, including 101 T2D patients with carotid atherosclerosis, could not confirm a significant anti‐inflammatory effect by semaglutide [141]. Moreover, in a study by Kataoka et al. [142] comparing atherosclerotic plaques in statin‐treated T2D subjects with and without GLP‐1RA using NIRS/IVUS, GLP‐1RA was only associated with reduced plaque lipid core burden.

SGLT2i have been shown to reduce cardiovascular death, all‐cause death, and hospitalization for heart failure. Interestingly, the most potent effects of SGLT2i are observed among individuals with heart failure, and the potential anti‐atherosclerotic effects in humans are less obvious according to the EMPA‐REG outcome trial [143] and the DECLARE‐TIMI 58 trial [144]. However, OCT‐imaging studies have shown that SGLT2i‐treated T2D individuals have a thicker fibrous cap, a smaller lipid core, and less inflammation [145, 146]. Moreover, in a longitudinal coronary CT angiography study including 236 patients with T2D and coronary atherosclerosis, SGLT2i was shown to reduce progression of non‐calcified plaques [147].

As T2D is a progressive disease, the endogenous insulin production may gradually decline. Over time, this decline can lead to the need for insulin‐replacement therapy, and the effects of exogenous insulin on cardiovascular complications have been debated. Herman et al. [148] reviewed the available evidence up to 2017 regarding the risk of insulin treatment and cardiovascular complications and found that most studies indicate that insulin therapy is associated with an increased risk of cardiovascular morbidity and mortality in T2D. Importantly, this risk seems to be dependent on the dosage and treatment length [149]. The risk can be related to the risk of hypoglycemia, iatrogenic hyperinsulinemia, and weight gain secondary to insulin usage.

### Lipid‐lowering therapies

The potent effect of statin treatment in preventing cardiovascular events in T2D was shown already in 2004 by the Collaborative Atorvastatin Diabetes Study and is today a well‐established part of the clinical guidelines [150]. As statin treatment may also stabilize plaques, the increased use of statins could potentially explain the reduced inflammatory activity and oxLDL accumulation observed in T2D plaques [53]. The addition of newer lipid‐lowering drugs, ezetimibe and PCSK9 inhibitors, now also allows us to reduce the circulating lipoprotein levels and cardiovascular risk even further [151, 152]. Even though the cardiovascular risk reduction seen with PCSK9 inhibitors may not be T2D‐specific, ODYSSEY DM‐DYSLIPIDEMIA showed that PCSK9 inhibition also reduced the risk of cardiovascular events among patients with T2D and dyslipidemia [153, 154].

In addition, icosapent ethyl has also been shown to affect plaque remodeling and plaque growth [155]. The REDUCE‐IT trial showed that treating individuals with high cardiovascular risk (CVD or diabetes and other risk factors) and high fasting triglyceride levels with icosapent ethyl significantly reduced the risk of cardiovascular events [156]. Icosapent ethyl affects triglyceride levels, but its protective effects in atherosclerosis have been suggested to be mediated through other mechanisms, including anti‐oxidative and anti‐inflammatory effects [157].

## Future treatment strategies—What could we target?

### Mechanism‐targeted therapies

As indicated by recent follow‐up studies, additional processes likely need to be targeted to further reduce the risk of cardiovascular complications in T2D. As T2D influences several mechanisms important in plaque formation and progression, such as oxidative stress and tissue repair, mechanism‐based therapies could be a potential therapeutical approach.

### Oxidative stress

Studies using antioxidants to treat diabetes‐associated vascular damage have shown varying results [158]. Systemic administration of exogenous antioxidants, such as dietary supplements, has not shown a vascular‐protective effect in subjects with diabetes [158]. Yet, when using a targeted‐therapy approach with “drug carriers” such as nanoparticles, results have been promising in animal models. Work by Wang et al. [159] and Mocanu et al. [160] has shown that blocking P‐selectin—an EC receptor that is important for monocyte migration—with nanoparticles did have an ROS‐lowering effect. The variability of the outcome data between systemic and targeted treatment strategies may be explained by the fact that a targeted therapy enables mechanism and/or cell‐specific delivery, thereby evading the “off‐target” effect.

The ROS generative enzyme NOX has been suggested to enhance oxidative stress in diabetes‐associated atherosclerosis based on in vitro and in vivo experiments. In support for this, inhibition of NOX1–NOX4 has been shown to be atheroprotective. Clinical studies have been conducted using the NOX1–NOX4 inhibitor setanaxib as a treatment in different disease settings, such as idiopathic pulmonary fibrosis [161], but no cardiovascular outcome trials are running yet. Apart from NOX1/NOX4, NOX5 has been implicated in both atherosclerosis [162, 163] and diabetes [164], making it another targetable NOX enzyme. Xanthine oxidase (XO) is also an enzyme that induces oxidative stress and has been found to be overexpressed in diabetes‐associated renal damage [165] and recently also shown to be associated with reduced CVD in subjects with cardiovascular risk factors [166]. Yet preclinical and clinical studies are needed to further understand the roles of these enzymes and their potential therapeutic potential.

### Inflammation

Even though recent studies of human plaques have not confirmed a greater inflammatory activity in T2D, inflammation remains a key factor in plaque formation and progression, particularly in the early stages of the disease. Moreover, enhanced inflammation likely drives fibrous cap thinning and necrotic core growth in established plaques.

The importance of inflammation as a key factor in atherosclerosis has been supported by studies such as COLCOT [167], LoDoCo2 (colchicine trials) [168], and the Canakinumab Anti‐inflammatory Thrombosis Outcomes Study (CANTOS; interleukin‐1β blockade) [169]. A subgroup analysis from the CANTOS trial showed that the preventive effects of interleukin‐1β blockade were similar among individuals with T2D compared to those without diabetes [170]. In line with this, a recent meta‐analysis also showed a lower risk of major adverse cardiovascular events among individuals with T2D receiving colchicine or canakinumab (four studies on colchicine and one on canakinumab) compared to placebo [171]. Moreover, there are other ongoing clinical trials, such as the Ziltivekimab Cardiovascular Outcomes Trial [172], aiming to target inflammation through IL‐6 inhibition. IL‐6 inhibition with ziltivekimab has been shown, among patients with elevated C‐reactive protein and chronic kidney disease, to reduce markers of inflammation and thrombosis. Despite that no comparison based on diabetes status was performed, 71% of all participants had diabetes [173]. Future studies will reveal whether this could be a promising target in T2D.

### Tissue repair

Plaques with thin fibrous caps are more common in T2D patients, as shown by histological and imaging studies [28, 35, 46, 100, 108]. Our group has recently shown that the thin fibrous caps in human T2D carotid plaques could be explained by an impaired interplay between contractile and fibroblast‐like VSMCs due to TGF‐β2 deficiency, which, in turn, affects collagen production [28]. We have also shown that stimulating key marker genes of the fibroblast‐like VSMC phenotype could contribute to collagen synthesis, which could potentially open up for a local therapeutic approach to stabilize plaques with a thin fibrous cap [174]. However, further studies are needed to explore if targeting fibrous cap formation could be a promising approach for plaque stabilization.

### Efferocytosis

Efferocytosis, the uptake and clearance of apoptotic cells, is another mechanism implicated in T2D‐associated atherosclerosis. Studies on efferocytosis using human models are sparse, yet theoretically, efferocytosis is an attractive therapeutical target. Interestingly, a study from 2025 did show that silencing IRF5 by nanoimmunotherapy was a promising treatment of atherosclerosis in ApoE^−/−^ mice [75]. Moreover, nanotherapeutical inhibition of CD47, an efferocytosis inhibitor acting as “don't eat me” molecule, has also shown promising results in both murine [175] and porcine [176] atherosclerosis models. However, whether these specific pathways would be promising targets to reduce T2D‐associated CVD in humans remains unknown.

### Epigenetic drugs

Inhibition of enzymes associated with epigenetic mechanisms such as DNA methylation and posttranslational modifications, thereby regulating the gene expression of disease‐related genes, is another potential therapeutic target. Apabetalone is an epigenetic modulator inhibiting bromodomain and extraterminal (BET) proteins and is one of the oral drugs that has gained attention in recent years. Apabetalone has been shown in both experimental and clinical studies to have beneficial cardiovascular effects [177, 178, 179]. Ray et al. [180] further investigated the effect of apabetalone on diabetes‐associated CVD, using a randomized control study design. A number of 2425 subjects with T2D and recent acute coronary event were randomized to apabetalone or placebo in addition to standard care. The study showed no significant risk reduction regarding primary endpoints (CV death, myocardial infarction, and stroke) but a decrease in congestive heart failure‐related hospitalization.

## Future perspectives

**Fig. 5:**
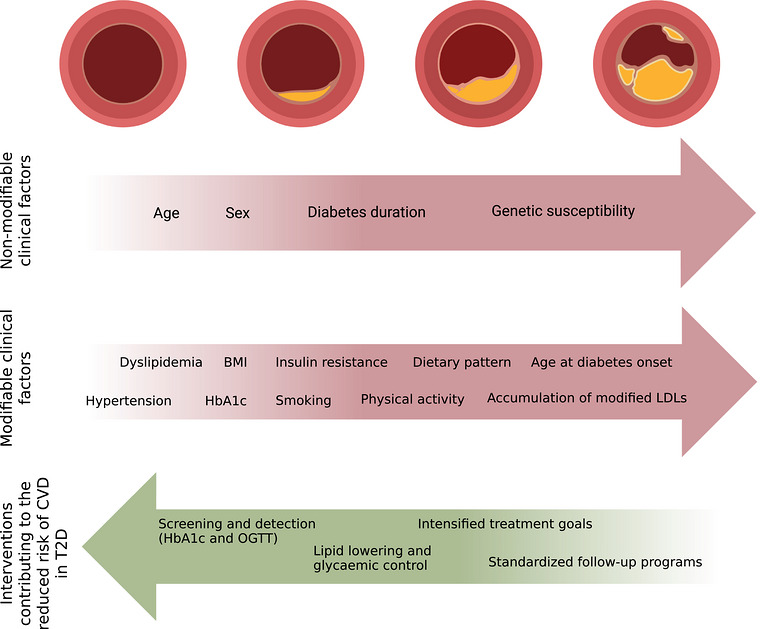
The changing role of type 2 diabetes as cardiovascular risk factor. The red arrows include the non‐modifiable and the modifiable clinical factors that contribute to the formation/progression of atherosclerosis and cardiovascular events. The green arrow indicates targeted factors that have influenced the risk of type 2 diabetes as a cardiovascular risk factor. BMI, body mass index; HbA1c, hemoglobin a1c; LDL, low density lipoprotein; OGTT, oral glucose tolerance test T2D, type 2 diabetes Created in BioRender. Al‐Sharify, D. (2026) https://BioRender.com/unb52tg.

T2D and its macrovascular complications are a major health burden worldwide. As research has advanced, new treatments have been introduced to the clinic, contributing to a decline in the incidence of T2D‐related CVD. Yet, despite this progress, more work remains to be done. Recent research indicates that T2D is a more complex disease than previously thought, and factors—such as the clinical phenotype and the genetic background—may need to be considered when studying T2D atherosclerosis. Targeting clinical risk factors and having efficient screening strategies are key to preventing atherosclerotic complications in T2D, but these interventions are not enough; new therapies are needed to reduce the risk even further. Moreover, when aiming to identify biological processes to target therapeutically, it is important to consider the time frame as the improved clinical treatment strategies affect the underlying biology (Fig. 5). Of note, advances in technology and methodology have increased our understanding of the biological changes in T2D‐associated atherosclerosis, yet more studies are needed to explore how these changes could be translated into therapies. Together with improved imaging, this will help us to individualize treatments to efficiently prevent atherosclerotic complications due to T2D.

## Author contributions

**Dania Al‐Sharify**: Conceptualization; writing—original draft; visualization; writing—review and editing. **Andreas Edsfeldt**: Conceptualization; writing—original draft; funding acquisition; writing—review and editing; visualization, resources, supervision. **Jiangming Sun**: Conceptualization; writing—original draft; visualization; writing—review and editing; supervision.

## Conflict of interest statement

Andreas Edsfeldt reports consulting fees from Novo Nordisk, Sanofi, Amarin, and Amgen, but this has not had any relationship with the current study or affected the design/outcome of the study.

## Funding information

Hjärt‐Lungfonden (Nos.: 20220044, 20220284, 20240143, and 20241210); Svenska Sällskapet för Medicinsk Forskning (No.: CG‐22‐0254‐H‐02); Vetenskapsrådet (No.: 2024‐02761); The Swedish Stroke Association (No.: S‐993166); Hjelt Foundations; Skånes universitetssjukhus; Stiftelsen Bundy Academy; Knut och Alice Wallenbergs Stiftelse; and Medicinska Fakulteten, Lunds Universitet.

## Data Availability

Data sharing not applicable to this article as no datasets were generated or analyzed during the current study.
